# Deep Learning-Driven Intelligent Fluorescent Probes: Advancements in Molecular Design for Accurate Food Safety Detection

**DOI:** 10.3390/foods14173114

**Published:** 2025-09-05

**Authors:** Yongqiang Shi, Sisi Yang, Wenting Li, Yuqing Wu, Weiran Luo

**Affiliations:** 1 Agricultural Product Processing and Storage Lab, School of Food and Biological Engineering, Jiangsu University, Zhenjiang 212013, China; 2 School of Grain Science and Technology, Jiangsu University of Science and Technology, Zhenjiang 212003, China

**Keywords:** deep learning, fluorescent probes, food safety, molecular design, inverse design, signal processing, pattern recognition, intelligent sensing

## Abstract

The complexity of global food supply chains challenges public health, requiring advanced detection technologies beyond traditional lab methods. Fluorescent sensing, known for its sensitivity and quick response, is promising for food safety but hindered by inefficient probe design and difficulties in analyzing complex signals in food. Deep Learning (DL) offers solutions with its nonlinear modeling and pattern recognition capabilities. This review explores recent advancements in DL applications for fluorescent sensing. We explore deep learning methods for predicting fluorescent probe properties and generating fluorescent molecule structures, highlighting their role in accelerating high-performance probe development. We then offer a detailed discussion on the pivotal technologies of deep learning in the intelligent analysis of complex fluorescent signals. On this basis, we engage in a thorough reflection on the core challenges presently confronting the field and propose a forward-looking perspective on the future developmental trajectories of fluorescent sensing technology, offering a comprehensive and insightful roadmap for future research in this interdisciplinary domain.

## 1. Introduction

Ensuring food safety is the cornerstone of maintaining global public health security, promoting sustainable economic development and maintaining social stability [1]. With the deepening of globalized trade and the wide application of modern agricultural technologies, the potential risk factors in the food supply chain have become increasingly diversified and hidden, covering every link from farm to table, including persistent organic pollutants (POPs), pesticide and veterinary drug residues, heavy metal ions, fungal toxins, illegal additives, as well as food-borne pathogenic microorganisms [2,3,4,5]. Traditional food safety standard testing methods, such as gas chromatography–mass spectrometry (GC-MS) and liquid chromatography–mass spectrometry (LC-MS/MS), although known as the “gold standard” with high sensitivity and accuracy [6,7], are expensive in terms of instrumentation and equipment, suffering from centralization limitations that fail to meet the urgent needs of modern society for large-scale, high-throughput, on-site and even in-line real-time monitoring [8,9].

**Figure 1:**
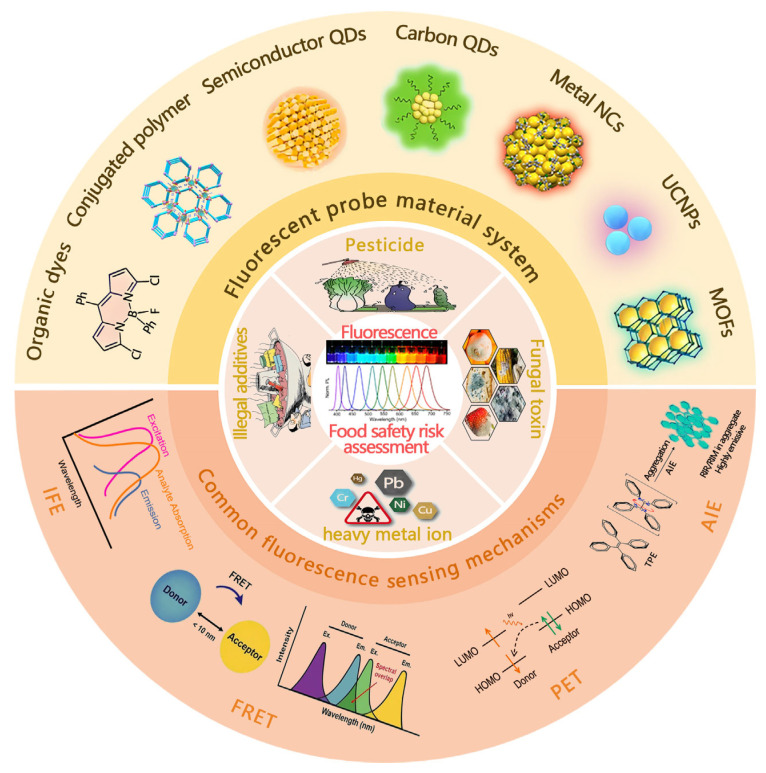
Fluorescent sensing materials (yellow), mechanisms (pink), and detection of typical food hazards (light red).

To address the limitations of traditional methods, various rapid detection techniques have been developed [10,11,12], among which fluorescence sensing analysis has stood out as one of the most dynamic research areas [13,14,15]. The technique utilizes measurable changes in the probe photophysical properties (e.g., fluorescence intensity, wavelength, lifetime, polarization state) induced by the interaction of a target analyte with a fluorescent probe molecule to achieve detection [16,17]. Their core strengths are unparalleled sensitivity (theoretically up to the single-molecule level), a nanosecond response time, non-invasiveness, and visualization capabilities through fluorescence imaging [18,19]. Material systems for fluorescent probes have also undergone significant development (Figure 1), from the initial organic small-molecule dyes [20,21] to conjugated polymers [22,23,24,25], semiconductor quantum dots (SQDs) [26,27,28], fluorescent carbon dots (CQDs) [29,30,31,32], metal nanoclusters (NCs) [33,34,35], rare-earth-element-based upconversion nanoparticles (UCNPs) [36,37,38,39], and metal–organic frameworks (MOFs) [40,41], among others. In addition, common fluorescence sensing mechanisms include Förster resonance energy transfer (FRET) [42,43,44,45], the internal filtering effect (IFE) [46,47], photoelectron transfer (PET) [48,49], and aggregation-induced luminescence (AIE) [50], which provide a rich toolbox for different application scenarios [51,52].

However, despite the many achievements, fluorescence sensing technology has been plagued by two systematic bottlenecks on its way to truly widespread and reliable practical applications. First, the development of traditional probes is highly dependent on the “chemical intuition” and experience of chemists [53,54], through the iterative cycle of “hypothesis–synthesis–test–correction”, which is a trial-and-error approach [55,56]. In the face of thousands of food safety hazards and increasingly stringent requirements on probe performance (e.g., near-infrared emission to penetrate biological tissues, high selectivity for specific ions, high quantum yield in aqueous phases), it is a great challenge for synthetic chemistry and materials science to move away from serendipitous discovery and achieve the functionally oriented and predictable rational design of molecules [57]. Secondly, food samples are known to be “dirty” matrices containing endogenous substances such as proteins, fats, pigments, vitamins, etc., which can cause background fluorescence, absorption or scattering interference [58], which is difficult to avoid [59,60] despite the fact that ratiometric fluorescent probes have been designed to reduce the matrix interference effect [61,62,63]. In addition, when multiple structurally similar analytes coexist, the probes tend to exhibit cross response, making it difficult to distinguish between analyses of a single signal dimension [64,65]. These issues can lead to compromised accuracy, context-dependent reproducibility, and elevated false-positive rates under demanding field conditions in practical applications [66].

In this review, the rise of deep learning (DL) brings a historic opportunity to break through the above dual dilemmas. As a branch of machine learning, deep neural networks are capable of constructing deep and complex nonlinear models, automatically learning and extracting abstract features from massive and high-dimensional data [67,68], and their success in fields such as image recognition and natural language processing has proved their powerful cognitive and creative abilities [69,70,71]. For the mathematical framework of deep learning, readers may refer to the article by Shlezinger et al. [72]. In recent years, DL has begun to penetrate into the field of basic science, showing great potential to reshape the paradigm of scientific discovery [73,74,75]. Although there have been reviews exploring various applications of fluorescent probes in food testing [76,77,78] or outlining the role of machine learning in analytical chemistry [79,80,81], respectively, they tend to suffer from perspective limitations. Most of the literature views deep learning only as a more powerful chemometrics tool at the end of the analytical process for data classification or regression [82,83,84], ignoring its revolutionary potential in the upstream of scientific discovery—i.e., the molecule creation stage.

**Figure 2:**
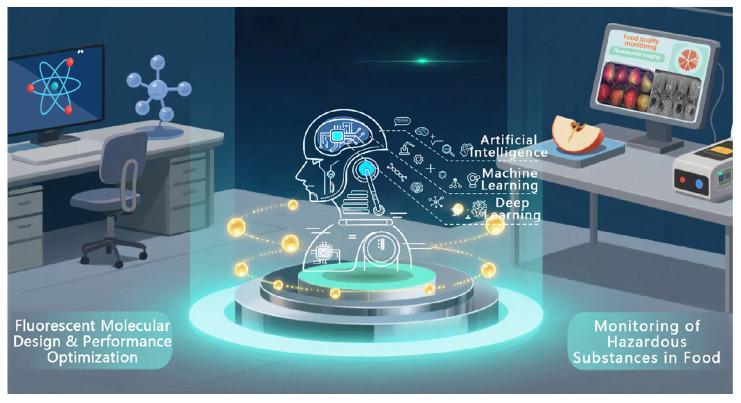
Deep learning enables molecular design for precision food safety detection through “forward prediction” and “reverse generation”.

The purpose of this review is to systematically elaborate the new paradigm of “Intelligent Design (e.g., DL-enabled molecular design and optimization)→Intelligent Sensing (e.g., AI-powered signal acquisition and decoding)→Intelligent Analyses (e.g., deep learning-based feature extraction and pattern recognition)”, which is a closed-loop approach with an integrated analytical pipeline that is carried out by deep learning. In this paper, we will focus on how deep learning can fundamentally change the discovery mode of fluorescent probes through “forward prediction” and “reverse generation”, and how deep learning can achieve the accurate decoding of complex and dynamic fluorescent signals through deep feature extraction and spatio-temporal pattern recognition (Figure 2). In the end, this paper aims to provide a systematic framework for the future development of smart sensing technology with comprehensive analytical capabilities for researchers in the interdisciplinary fields of chemistry, materials, analytics, food, and computer science through the systematic consideration of current challenges and future development directions.

## 2. Deep Learning for Fluorescent Probe Design: From Screening to Generation

The performance of fluorescent probes is rooted in their molecular structure. Deep learning is transforming probe design from an “art” to a computational “science” by constructing high-precision mappings between structures and properties, making the leap from mass screening to on-demand creation.

### 2.1. Property-Driven Design: DL-Based QSAR and Virtual Screening

The core task of “Forward Design” is to rapidly and accurately predict a set of key properties of a molecule given its structure. For fluorescent probes, these critically include absorption/emission wavelengths (λ_abs_/λ_em_), fluorescence quantum yields, Stokes shifts, and photostability—properties that directly govern sensing performance. The accurate prediction of these parameters enables the rational design of probes with enhanced brightness, target specificity, and environmental stability. This is a prerequisite for high-throughput virtual screening (HTVS). Traditional QSPR (Quantitative Structure-Performance Relationship) methods rely on manually designed molecular descriptors (e.g., topological indices, physicochemical parameters), which often have incomplete and biased information [85,86]. Deep learning, particularly graph neural networks (GNNs), is changing this paradigm [83,87,88]. GNNs naturally represent molecules as graphs (atoms as nodes and bonds as edges), and iteratively aggregate information about neighboring atoms and bonds across the graph through a mechanism known as ‘message passing’, thus generating for each atom an iterative graph containing its localized information. Each atom generates a rich vector representation containing information about its local chemical environment and even global topology [89,90]. Ultimately, a molecular-level representation can be obtained by performing a pooling operation on all atom representations within the predefined molecular graph structure, which are then input into the fully connected layer for property prediction.

A variety of state-of-the-art GNN architectures, such as message-passing neural networks (MPNNs) [91], SchNet [92], and DimeNet/DimeNet++ [93] (which can explicitly encode interatomic distance and angle information, which is more physically intuitive), have proved to be highly successful in predicting molecular properties. Using publicly available databases containing hundreds of thousands of molecules (e.g., QM9 [94], ZINC [95]) or self-constructed specialized databases, researchers have trained GNN models to predict the core photophysical parameters of fluorescent probes. For example, by training on large fluorescent dye databases (e.g., FluoDB or privately constructed databases), the GNN model is able to predict the molecule’s maximum absorption/emission wavelengths (λ_abs_/λ_em_), molar absorbance coefficients, fluorescence quantum yields, Stokes shifts, and even two-photon absorption cross-sections with accuracies comparable to or exceeding those achieved by conventional density-functional theory [96,97], particularly for certain properties, while acknowledging that DFT retains unique advantages in providing fundamental physical insights.

**Figure 3:**
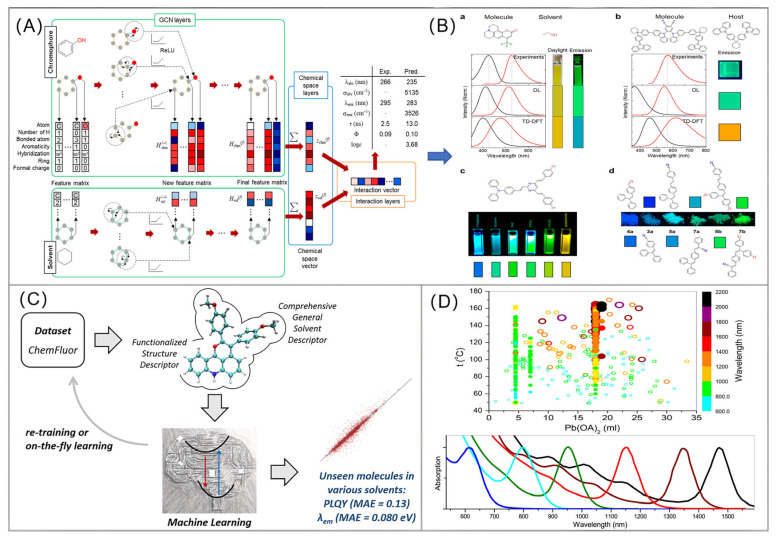
(**A**) A graph neural network (GNN) model-coupled ligand solvent interaction used to predict the optical and photophysical properties of ligands. The figure was reproduced from [98], with permission from American Chemical Society, 2021; (**B**) Experimentally measured and DL predicted absorption (black) and emission (red) spectra. (a) Coumarin 153 in ethanol. (b) BPCP-2CPC (molecule) in C-2PC (host). The bandwidths in fwhm for the calculated spectra are set to 5370 cm^−1^. (c) Photograph of (E,E,E) -2-(4-diphenylaminostyryl) -4,6-bis (4-methoxystyryl) pyrimidine in several solvents. The colors below the photograph are those predicted using the proposed GNN model from (**A**). (d) Photographs of solid state emission. The colors below the photograph are those predicted using the proposed DL model from (A). The figure was reproduced from [98], with permission from American Chemical Society, 2021; (**C**) A different (non-GNN) machine learning model was used to predict the emission wavelength and quantum yield of organic fluorescent materials. The figure was reproduced from [99], with permission from American Chemical Society, 2021; (**D**) Utilizing machine learning (non-GNN) to optimize synthesis parameters of colloidal quantum dots. The figure was reproduced from [100], with permission from American Chemical Society, 2019.

Sungnam Park et al. introduced a deep learning model based on GNN for chromophore–solvent interactions (Figure 3A), which accurately and reliably predicted the optical and photophysical properties of organic compounds (Figure 3B) [98]. Cheng-Wei Ju et al. established a database containing over 4300 solvated organic fluorescent dyes and developed a new machine learning method (non-GNN) to predict emission wavelengths and photoluminescence quantum yields (PLQY) (Figure 3C). Evaluation using unseen molecules showed that this model is comparable to time-dependent density functional theory (TD-DFT) calculations [99]. Voznyy et al. utilized machine-learning-in-the-loop (non-GNN) to learn from available experimental data [100] and propose experimental parameters to try, and, ultimately, point to regions of synthetic parameter space that will enable record-monodispersity PbS quantum dots (Figure 3D). Additionally, its prediction speed is improved by five to seven orders of magnitude compared to DFT calculations, from hours/days to milliseconds [101]. This amazing efficiency makes comprehensive screening in a chemical space containing hundreds of millions or even billions of virtual compounds a reality [102]. In addition, DL models can predict the binding affinity of molecules to specific targets (e.g., protein active sites, nucleic acid sequences) [103,104], enabling truly functionally oriented design [105].

### 2.2. Structure-Driven Design: Generative Models for On-Demand Probes

If forward design is a “needle in a haystack”, then Inverse Design is the important goal in molecular discovery—generating completely new molecular structures that meet the requirements based directly on a set of predefined target properties [106,107]. Deep generative modelling is the core engine to achieve this goal.

**Figure 4:**
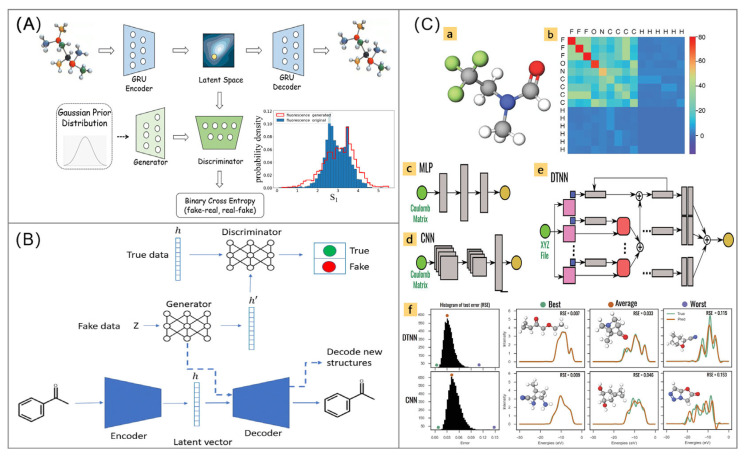
(**A**) De novo creation of fluorescent molecules via adversarial generative modeling. The figure was reproduced from [112], with permission from Royal Society of Chemistry, 2023; (**B**) LatentGAN which combines an autoencoder and a generative adversarial neural network for de novo molecular design. The figure was reproduced from [113], with permission from the BMC, 2019; (**C**) Predicting molecular excitation spectra based on Machine learning and deep learning models. (a) Atomic structure of the N-methyl-N-(2,2,2-trifluoroethyl)formamide molecule and (b) its corresponding Coulomb matrix representation. Canonical illustration of the three neural network types: (c) the multilayer perceptron (MLP); (d) the convolutional neural network (CNN); and (e) the deep tensor neural network (DTNN). Green circles to the left represent the molecular input and yellow circles to the right the output (here 16 excitation energies or the molecular excitation spectrum). The gray blocks are schematics for fully connected hidden layers, convolutional blocks, pooling layers, and state vectors. Nodes corresponding to atom types in the DTNN are represented as blue squares and the distances matrix between different atoms as pink squares. Parameter tensors (red squares) project the vectors encoding atom types and the interatomic distance matrix into a vector with same dimensions as the atom type encodings. The DTNN is evaluated iteratively, building up more complex interactions between atoms with each iteration. (f) Comparison of CNN and DTNN spectra predictions: the first column depicts RSE histograms for 13 000 test molecules from the 132k dataset. The following three columns show the spectra of the best, an average, and one of the worst predictions compared to the corresponding reference spectrum. The colored circles mark the histogram positions of the selected molecules. The figure was reproduced from [114], with permission from Wiley, 2019.

Variational Autoencoders (VAEs) are one of the key classes of techniques. A typical molecular VAE consists of an encoder and a decoder [108]. The encoder compresses the discrete molecular map (or SMILES string) into a continuous, low-dimensional “latent space” that satisfies a specific probability distribution (usually Gaussian). The decoder then reconstructs the molecular structure from a point (vector) in this latent space. The structural characteristics of this latent space are its continuity and structure: molecules with similar properties have similar positions in the latent space [109]. By training the property predictor in conjunction with the VAE, it is possible to make certain dimensions of the latent space correlate with specific molecular properties (e.g., emission wavelength). Gradient up optimization can then be performed in the latent space in the direction that optimizes the target property (e.g., maximizing the quantum yield while fixing the emission wavelength at 700 nm), and the optimized vectors of the latent space can then be fed into the decoder to generate a completely new molecule with the desired property [110,111]. Critically for fluorescent probes, this requires embedding photophysical constraints like Stokes shift thresholds and aqueous stability during optimization. Shi et al. employed an autoencoder-based generative adversarial network (AGAN) to generate molecular SMILES. AGAN conducts unified training by simultaneously optimizing the encoder/decoder and the generative adversarial network (Figure 4A). The results show that the excited-state property distribution of the generated molecules completely matches the original samples, and the feasibility of molecular synthesis is also achieved [112].

Another powerful class of generative models is Generative Adversarial Networks (GANs) [115], which contain a Generator and a Discriminator. The Generator attempts to create chemically valid and authentic-looking molecules from random noise, while the Discriminator endeavors to distinguish between real molecules (from the training set) and molecules faked by the Generator. The two co-evolve in adversarial training, eventually forcing the generator to master the basic rules of chemical structure and be able to create novel and diverse molecules [116]. When generating fluorescent probes, GANs must additionally satisfy spectral criteria (e.g., excitation/emission gap > 100 nm) and avoid known quenching motifs. Johansson et al. proposed a new deep learning architecture, LatentGAN, which combines autoencoders and generative adversarial neural networks for de novo molecular design (Figure 4B). The results show that the compounds sampled by the trained model can largely occupy the same chemical space as the training set and can generate a large number of novel compounds [113]. Rinke et al. trained and evaluated three different neural network architectures—Multilayer Perceptron (MLP), CNN and Deep Tensor Neural Network (DTNN)—using the electronic energy level density of 132,000 organic molecules as an example, for predicting molecular excitation spectra [114] (Figure 4C). For fluorescent probe design, such predictions must account for solvent-dependent spectral shifts and aggregation effects. These studies provide references for the generation of functional fluorescent molecules.

To achieve goal-directed generation, Reinforcement Learning (RL) can be combined with GANs [117]. In this framework, the generator is considered as an “agent” whose “action” is to build the molecule step by step (e.g., adding atoms and bonds one by one). After each step, the agent receives a “reward” if the resulting intermediate structure or final molecule is closer to the target property, as evaluated by the QSPR model. By maximizing the cumulative reward, the generator learns how to strategically construct structures to meet complexity performance requirements [118,119]. In fluorescent probe RL, reward functions typically penalize structures prone to PET quenching or with poor photostability. In addition, Flow-based models [120] and Diffusion models [121] are emerging as newer generative techniques for molecular design because of their ability to generate high-quality, diverse molecules and more stable training processes. These “on-demand” methodologies imply a shift in chemical discovery from a passive “screening” mode to an active “creation” mode, providing a potential rapid response to novel and unknown food safety threats. A comparison of these model architectures’ advantages and challenges is provided in Table 1.

## 3. Intelligent Fluorescent Signal Processing and Feature Extraction

Designing the ideal probe molecule is the first step in building a high-performance sensing system. The second, and equally critical, step is to extract the weak but valuable analytical signals from the real, complex sample environment. Deep learning plays the role of “smart decoder” in this process, with capabilities far beyond those of traditional chemometrics.

### 3.1. Matrix Interference Correction: Signal Enhancement and Quantification

The complexity of food matrices is a major impediment to the practical application of fluorescence analysis. Casein and fat globules in milk, pigments and phenolics in fruit juices, and myoglobin in meat extracts produce strong autofluorescence, Rayleigh/Raman scattering, and severe Inner Filter Effect (IFE), which interferes with interferences that are often several orders of magnitude stronger than the target signals, leading to the risk of invalidating quantitative methods based on peak height or peak area [123,124]. For fluorescent probes, this interference disproportionately affects probes with small Stokes shifts or short lifetimes, demanding specialized correction approaches. Deep learning models, especially convolutional neural networks (CNNs), provide powerful solutions to this challenge. For one-dimensional fluorescence spectra, they can be treated as one-dimensional signals and processed using 1D-CNNs [125]. Through its multi-layer convolutional kernel, 1D-CNN is able to automatically learn and recognize multi-scale features in the spectrum [126,127], such as the characteristic peak shapes of the target, the statistical patterns of the noise, and the complex morphology of the baseline drift, without the need for manual pre-processing [128]. By end-to-end training on a dataset containing a large number of “interfering spectra + target concentration” pairs, the CNN can directly regress the exact concentration of the target from the original, heavily contaminated spectra, and its intrinsic nonlinear modelling capability allows it to implicitly learn and correct for a variety of complex matrix effects [129,130].

For the more informative three-dimensional fluorescence spectra, i.e., Excitation-Emission Matrix (EEM), 2D-CNN is able to bring out its great advantages in the image processing field [131]. EEMs can be directly treated as a two-dimensional image, where the target fluorescence peaks, Rayleigh/Raman scattering ridges, and background fluorescence regions present different shapes and texture features. 2D-CNN can efficiently capture these spatial features for the accurate segmentation and identification of different signal sources [132,133]. For example, by using semantic segmentation networks such as U-Net, researchers can accurately “key out” pure target fluorescent regions from EEMs and then quantify them to eliminate scattering and other fluorescent substances [134]. In addition, Autoencoders and their variants (e.g., noise-reducing autoencoders) have been widely used for spectral denoising and feature extraction [135], where the model is forced to learn the intrinsic features of the signal by compressing the spectrum into a low-dimensional “bottleneck” layer and then reconstructing it to efficiently filter out random noise and irrelevant information [136].

### 3.2. Fluorescent Probe Arrays: Chemical Fingerprinting with Deep Learning

In the face of multi-component mixtures with highly similar chemical structures (e.g., multiple carbamate pesticides, or honey from different origins), the development of a single selective probe often falls short of the task. A smarter strategy is to construct an array of multiple semi-selective (or cross-reactive) fluorescent probes, a “fluorescent chemotaxis” or “chemotongue”, borrowing from the biological olfactory and gustatory systems [137]. Each probe in the array produces a different level of fluorescence response when interacting with the analyte system, and the responses of all the probes combined form a unique high-dimensional “chemical fingerprint” of the sample [138,139].

Deep learning models, with their powerful nonlinear classification capabilities, are ideal tools for parsing these complex chemical fingerprints. The response vectors of the probe arrays can be directly fed into a fully connected dense convolutional network (DenseNet). Utilizing dense connections between layers encourages feature reuse and improve gradient flow, thereby enhancing its capacity to learn complex hierarchical representations from high-dimensional data, which is capable of finding complex decision boundaries in the high-dimensional space capable of distinguishing between different sample classes through a multilayered nonlinear transformation [138]. If the response signals of an array of probes are arranged into a two-dimensional matrix (e.g., rows representing different probes and columns representing responses at different excitation wavelengths), then they can be processed using CNNs [140]. The ability of the convolutional kernel of a CNN to automatically learn local patterns of synergistic responses between probes, as well as global response compositions, is essential for identifying the subtle “fingerprint” differences arising from multicomponent synergistic interactions [141].

**Figure 5:**
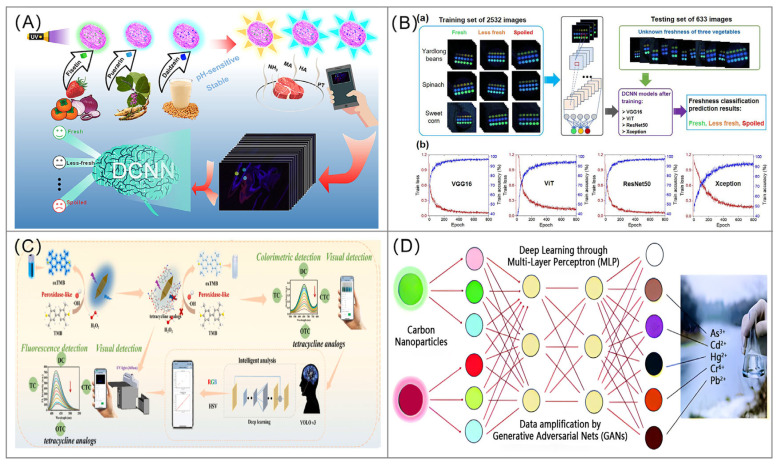
(**A**) Deep learning-assisted flavonoid-based fluorescent sensor array for the nondestructive detection of meat freshness. The figure was reproduced from [152], with permission from Elsevier, 2024; (**B**) Fluorescent sensor array combined with DCNN model for freshness prediction of three vegetables. (a) Flowchart of DCNN for freshness prediction. (b) Training loss and training accuracy of the four DCNN models decrease and increase with the increase of training epochs. The figure was reproduced from [153], with permission from Elsevier, 2024; (**C**) Nanozyme-induced deep learning-assisted smartphone integrated colorimetric and fluorometric dual-mode for detection of tetracycline analogs. The figure was reproduced from [149], with permission from Elsevier, 2024; (**D**) Generative Adversarial Nets (GANs) assisted detection of toxic heavy metal ions based on visual fluorescence responses from a carbon nanoparticle array. The figure was reproduced from [142], with permission from Royal Society of Chemistry, 2022.

As shown in Figure 5, researchers have successfully utilized probes arrays based on different fluorescent nanomaterials in combination with deep learning models to achieve the identification of a wide range of heavy metal ions [142,143,144,145], pesticide residues [146,147], amino acids [148], illegal additives [149,150,151], food freshness [130,152,153,154,155], food adulteration [156,157], and foodborne pathogens and their toxins [158,159]. Table 2 shows some typical cases of using fluorescent probes combined with deep learning or machine learning techniques for food analysis. The choice of specific deep learning architectures in these applications is driven by task requirements; for instance, ResNet-101 is often favored for its ability to extract deep hierarchical features from complex spectral or image data, while YOLO’s real-time object detection capability makes it highly suitable for tasks like dynamic freshness assessment. This ‘low-selectivity probe & high-intelligence algorithm’ strategy strategically couples computational intelligence with probe chemistry, where deep learning enhances spectral interpretation for multi-component systems while chemical design principles continue to govern probe development, thereby reducing demanding selectivity requirements for probes and opening new paths to solve thorny, multi-component food safety problems.

### 3.3. Kinetic Process Modeling: Nonlinear Quantification with RNNs

Many fluorescence sensing processes are inherently dynamic; this includes, for example, organophosphorus pesticide detection based on enzyme inhibition reactions, where the fluorescence signal may change with the gradual loss of acetylcholinesterase (AChE) activity [164], sensing based on conformational changes in a nucleic acid aptamer upon binding to a target [165], where the fluorescence resonance energy transfer (FRET) efficiency evolves over time [166]; and adsorption or catalytic processes between nanomaterials and analytes [167,168]. The kinetic profiles of these fluorescence signals over time contain far richer information than single endpoint readings, such as reaction rates, delay times, etc., and these dynamic features are crucial for distinguishing between different types or concentrations of analytes [169]. RNNs (especially LSTM/GRU) are uniquely suited to such tasks as they learn temporal patterns directly from sequence data, bypassing the need for predefined kinetic equations which often fail under complex nonlinearities or noise.

Recurrent Neural Networks (RNNs), particularly their advanced variants that overcome the gradient vanishing problem, Long Short-Term Memory Networks (LSTMs) [170,171,172] and Gated Recurrent Units (GRU) [173], are specifically designed to process and analyze sequence data. By taking fluorescence dynamics features as input time series, LSTM is able to capture the long-term dependencies and subtle trends of signals in the time dimension using its internal “memory units” [174]. Shi et al. [175] built an LSTM and radial basis function (RBF) based on optimized excitation–emission matrices (EEMs) from fisheye fluid. LSTM and radial basis function neural network (RBFNN) models were used to accurately predict the changes in rainbow trout freshness under non-isothermal storage conditions. In their work, Wei et al. utilized an edge-deployed multimodal nano-sensor array combined with a CNN-LSTM deep learning model to achieve the real-time monitoring of multi-pollutant water quality [176]. Separately, a key finding demonstrates that LSTM was able to accurately predict the final result based on the initial trend of the curve at an early stage when the reaction was far from equilibrium, thus reducing the detection time from hours to tens of minutes [177]. While powerful, these models demand significant computational resources for training and may lack interpretability compared to simpler kinetic models, posing challenges for real-time edge deployment or mechanistic studies. In addition, for sensing systems with highly nonlinear dose–response relationships, deep neural networks are able to fit these complex functional relationships with great accuracy, ensuring accurate quantification over the entire wide dynamic range [178].

## 4. Core Challenges, Deep Reflections and Future Scenarios

### 4.1. Current Core Challenges

1. The data gap and quality dilemma: The power of deep learning is rooted in large amounts of high-quality, well-labelled data. However, in the field of chemistry and sensing, access to such data is an extremely expensive “luxury”. Publicly available, standardised databases of fluorescent molecular properties are still limited in size and mostly restricted to basic photophysical parameters, lacking data directly related to sensing performance [179]. On the sensing application side, fluorescence response data involving real food matrices are even more scarce, heterogeneous, and difficult to reproduce. The construction of large-scale comprehensive databases that are open, shared, and follow the FAIR (discoverable, accessible, interoperable, and reusable) principle is the cornerstone for advancing the field. At the same time, it is also crucial to develop Few-shot Learning (FSL) to reduce the reliance on massive labelled data [180]. However, FSL performance remains sensitive to data distribution shifts and may struggle with highly nonlinear chemosensing relationships. In fluorescent probe design, this scarcity critically impedes predicting analyte-binding affinities and optimizing Stokes shifts—key parameters for rationetric sensing.

2. The contradiction between model interpretability (XAI) and scientific discovery: Deep learning models, especially deep networks, are opaque in their decision-making process, which is in tension with the scientific spirit of pursuing cause and effect and mechanism. In probe design, we not only want to know that an AI-designed molecule “works”, but also “why it works”, and what are its key structural motifs or mechanisms of action? In signal analysis, understanding which spectral features or probe response patterns the model is based on can help us discover new sensing mechanisms or optimise array design. Therefore, the development of interpretable AI techniques, such as the use of Attention Mechanism [181] to visualise the focus of a model’s attention, is essential to make the leap from “black-box” prediction to “white-box” insight, and thus to truly guide scientific innovation. Current XAI methods face challenges in providing chemically meaningful interpretations for complex nonlinear sensor responses. For fluorescence signal interpretation, this obscurity hinders identifying critical spectral bands or quenching pathways that govern target recognition specificity.

3. Generalisability and Domain Adaptation Challenge: A model trained in one lab, with one instrument, for one food matrix (e.g., plain milk), may suffer a sharp drop in performance when applied to another lab, with another instrument, or to another matrix (e.g., yoghurt, milk powder, juice). This is a typical Domain Shift problem. The development of more robust and generalisable model architectures, as well as the effective use of techniques such as Transfer Learning [182,183], which allows models to be rapidly “fine-tuned” and adapted with only a small amount of data from new scenarios, are key bottlenecks in the transition from “lab toys” to “industrial-grade tools”. Transfer learning efficacy is constrained when source-target domain gaps are large, particularly across different instrumentation or food matrices with varying interferents. In real-world probe deployment, matrix-induced fluorescence quenching or scattering effects frequently trigger catastrophic domain shifts beyond standard transfer learning capabilities.

4. Integration of physical and chemical laws: Purely data-driven deep learning models are “agnostic” in the sense that they do not have any a priori knowledge of physics or chemistry, and may sometimes make predictions that defy basic scientific principles (e.g., predicting negative quantum yields or generating chemically unstable molecules). How to embed known laws of physics and chemistry (e.g., Kasha’s rule, molecular orbital theory, reaction kinetics equations) as a strong constraint or regularisation term into the structural design or loss function of neural networks is an important direction to improve the prediction accuracy, data efficiency and extrapolation capability of the models [184,185]. However, mathematically formalizing complex chemical principles for model constraints remains challenging, and overly rigid physical embeddings may limit discovery of novel sensing mechanisms. Specifically for fluorescent probes, formalizing rules like Kasha’s Vavilov behavior or FRET distance constraints remains experimentally intractable for supramolecular systems, limiting rational design.

### 4.2. Future Development Landscape and Outlook

1. Application of Physics-Informed Neural Networks (PINNs): as a direct solution to the fourth challenge above, Physics-Informed Neural Networks (PINNs) [186] encode partial differential equations (e.g., equations describing the kinetics of mass transfer or reactions) directly into the loss function of the neural network, forcing the output of the model to satisfy the data fit while also having to obey the laws of physics. For the field of fluorescence sensing, this means that intelligent analytical models can be constructed that simultaneously fit experimental data and obey either fluorescence burst theory (e.g., the Stern-Volmer equation) or enzyme kinetic models (e.g., the Michaelis-Menten equation), resulting in more reliable and physically interpretable results. Specifically for fluorescent probes, PINNs can enforce: first, quantum yield ≥ 0.4 in aqueous media; second, förster distance constraints in FRET pairs; third, photobleaching kinetics ≤ 5%/min. However, practical implementation faces challenges such as the complexity of designing effective loss functions that balance data fidelity with physical constraints for intricate fluorescence phenomena, and the requirement for sufficient high-quality experimental data to train robust models.

2. Multimodal sensing information fusion and the rise of the Transformer architecture: by fusing information from different sensing modalities (e.g., fluorescence spectra, Raman spectra, mass spectrometry, electrochemical signals, hyperspectral images, etc.), a more comprehensive and robust characterisation of the sample can be obtained, thus overcoming the limitations of a single modality. The Transformer architecture, initially designed for natural language processing, thanks to its powerful long-range dependency capture capability and parallel processing advantages, has shown potential to outperform CNNs in areas such as computer vision [187,188]. Its application to processing multimodal sensing data is expected to extract deeper, cross-modal features, thereby significantly improving the accuracy and reliability of complex food system analysis. For fluorescence-centric fusion: first, time-resolved fluorescence decay (ns-μs) as core modality; second, cross-attention between fluorescence lifetime and Raman peaks; third, embedding solvent polarity effects in positional encoding. A key caveat is the current scarcity of large, well-annotated multimodal datasets specific to food fluorescence analysis needed for effective Transformer training, alongside the significant computational resources often required for these large models.

3. Edge Computing and Lightweight Model Diffusion: in order to achieve true on-site, real-time food safety monitoring, it is crucial to deploy complex deep learning models to resource-constrained portable devices or sensor nodes. This requires strong development of model compression techniques (e.g., knowledge distillation [189]), the design of lightweight network architectures, and the incorporation of Edge Computing frameworks [190], which enable data processing and intelligent decision making to be done close to the source of the data, thus lowering latency, preserving data privacy, and reducing the reliance on cloud computing resources. While edge deployment of intelligent sensing systems offers potential advantages for real-time monitoring, several technical challenges remain to be addressed. Current hardware constraints (e.g., computational capacity and energy efficiency) and the demand for model robustness in dynamic environments represent active research areas. Although advances in energy-efficient chipsets and adaptive learning algorithms may help mitigate these limitations, further validation of their performance in real-world scenarios is still required. Critical for fluorescence field deployment: first, real-time lifetime fitting on ≤100 mW processors; second, on-device correction of temperature-dependent Stokes shift; third, energy-efficient inference under probe photobleaching drift. Real-world deployment on edge devices necessitates careful trade-offs between model complexity/accuracy and the strict limitations of power consumption, memory, and processing capabilities inherent in portable hardware, which can impact the sophistication of the algorithms that can be reliably run.

## 5. Conclusions

Deep learning is systematically reshaping every aspect of fluorescence sensing technology from molecular design to signal analysis to practical applications with unprecedented depth and breadth. It not only improves the upper performance limit and R&D efficiency of the sensing system from the source by accelerating and empowering the rational design and ab initio creation of probes, but also significantly enhances the accuracy, robustness, and multitasking capability of fluorescence analysis in complex food matrices through its powerful pattern recognition, nonlinear modelling, and multi-dimensional information mining capabilities on the application side. Despite the challenges of data, interpretability, generalisation and physical consistency, with the continuous breakthroughs and integration of cutting-edge technologies such as physical information neural networks, multimodal fusion and edge AI, it is reasonable to believe that an AI-driven, high-throughput, high-precision food safety intelligent monitoring network will be realised, building a solid foundation for the protection of the people’s “safety on the tip of the tongue”. Building an impenetrable technological line of defence for the protection of people’s “safety on the tip of the tongue.

## Figures and Tables

**Table 1:** Comparison of typical model architectures’ advantages and challenges.

Model Architecture	Key Advantages	Key Challenges	Controllability/Goal Orientation	Seminal Paper
Variational Autoencoder (VAE)	Smooth latent space, facilitating gradient-based property optimization and interpolation.	Sometimes low validity of reconstructed molecules; tends to generate training set-similar molecules.	Good, via joint training with property predictors or latent space optimization.	[111]
Generative Adversarial Network (GAN)	Generates high-quality, novel, and diverse molecules.	Unstable training, prone to mode collapse.	Moderate, typically combined with RL or conditional GAN.	[115]
Reinforcement Learning (RL)-Guided Model	Directly optimizes complex, non-differentiable rewards (e.g., synthetic accessibility); strong goal orientation.	Difficult reward function design; hard to balance exploration/exploitation.	Very high, enables multi-objective optimization via well-designed rewards.	[117]
Diffusion Model	Capable of generating extremely high-quality samples, exhibiting good diversity, stable training dynamics.	Slow sampling (due to iterative denoising process), large model size requirements, high computational cost per sample.	Good, achievable via guidance or conditional input.	[121]
Flow-based Model	Exact and efficient likelihood calculation, enabling precise probability density estimation; invertible transformations, stable training.	Strong architectural constraints (e.g., requiring bijective transformations), potentially high computational cost during training/inference.	Moderate, achievable via conditional flow models.	[122]

**Table 2:** Performance comparison of fluorescent sensor arrays combined with deep learning or machine learning techniques in food analysis.

Sensor Array Composition/ Probe Material	Food Matrix	Target Analyte(s)	Machine Learning/ Deep learning Model	Key Performance Metrics	Reference
Copper nanoclusters (CuNCs) & fluorescent dyes	Pork	Meat freshness (Ammonia, dimethylamine, trimethylamine)	SqueezeNet (CNN), Grad-CAM, UMAP	Limit of detection (LOD): 131.56 ppb. Accuracy: 98.17%.	Lin et al. [130]
EuMOF-FITC	Fish products	Fish freshness	ResNext-101	LOD: 3.94 ppm (NH_3_). Accuracy: 98.97%.	Xu et al. [140]
Cys/NAC–AuNC&3D fluorescence spectra	Foods	Vitamin B6 derivatives	DNN, CNN	Accuracy: 97.77–100%. R^2^ = 97.01%	Noreldeen et al. [141]
Flavonoid-based fluorescent sensor array	Packaged meat	Meat freshness	DCNN	Accuracy: 97.1%.	Li et al. [152]
Rhodamine B-CD@Au, rhodamine 6G-CD@Au, & coumarin 6-CD@Au	Vegetables and fruits	Pyrethroid pesticides (PPs)	HCA, SVM, BPNN	LOD: magnitude of ppm. Recovery: 94.7–105%.	Li et al. [146]
Carbon dot & europium-doped calcium fluoride	Milk, egg	Tetracycline antibiotics (TCs)	Resnet18	LOD: 0.05 μM. Accuracy: 99.0%.	Chen et al. [160]
Au NCs@ Fe-MIL-88NH_2_	Water samples	Hg^2+^ and thiram	Yolov3	LOD: 7 nM (Hg^2+^). Precision: 97.1%.	Lu et al. [161]
Carbon dots (CDs) &Ru-MOFs	Shrimp and pork	Food freshness	YOLO	Recovery: 98.63–106.64%, RSD < 1.56%.	Wu et al. [162]
Carbon dots & CdTe quantum dots	Foods	Nine antibiotics	SX-model	Accuracy: 95%. average concentration error for unknown samples: 4.93%	Xu et al. [163]
